# Evaluating the repurposing potential of the anti‐depressant Agomelatine for Alzheimer’s Disease

**DOI:** 10.1002/alz.089356

**Published:** 2025-01-03

**Authors:** Grace A Terry, Michelle Merav, Patricia Rockwell, Peter Serrano, Lei Xie, Maria Figueiredo‐Pereira

**Affiliations:** ^1^ Hunter College, New York, NY USA; ^2^ Weill Cornell Graduate School of Medical Sciences, New York, NY USA; ^3^ The Graduate Center, CUNY, New York, NY USA

## Abstract

**Background:**

The limited treatment options for Alzheimer’s emphasizes the need to explore novel drug targets and bring new therapeutics to market. Drug repurposing is an efficient route to bring a safe and effective treatment to the clinic. Agomelatine (AGO) was identified by a high‐throughput drug screening algorithm as having mechanistic potential to treat Alzheimer’s. AGO is used as an atypical antidepressant and works as an MT1/MT2 receptor agonist and a 5HT2C serotonin receptor antagonist.

**Method:**

The TgF344‐AD rat model was used to test AGO’s potential to reduce cognitive deficits and neuropathology. The TgF344‐AD rat model expresses human mutant “Swedish” amyloid‐precursor protein (APPsw) and a Δ exon 9 presenilin 1 (PS1ΔE9). As it presents with age‐dependent progressive Alzheimer’s pathology and cognitive decline it is an ideal model for investigating AGO’s effect on the robust presentation of Alzheimer’s. Treatment with AGO at ∼10 mg/kg body weight/day began at 5 months of age (pre‐pathology) and continued until 11 months of age when cognitive testing (active place avoidance task) and tissue collection occurred. Immunohistochemistry was used to evaluate amyloid beta plaque burden. Bulk RNAsequencing was conducted to investigate AGO’s effect on gene expression.

**Result:**

AGO treated female TgF344‐AD rats showed reduced cognitive deficits with an increased latency to first entrance in aPAT testing compared to non‐treated transgenic littermates. There were no differences between the cognitive performance of AGO treated and untreated male TgF344‐AD rats. Interestingly, this reduced cognitive deficit did not correlate with decreased amyloid beta pathology. RNA sequencing analysis showed that DDIT3 (CHOP) mRNA levels were downregulated in the AGO treated compared to untreated TgF344‐AD females. DDIT3 is a pro‐apoptotic transcription factor.

**Conclusion:**

Agomelatine showed a female only reduction in cognitive deficits, which did not correlate with a decrease in amyloid beta plaque deposition. This finding paired with the decrease of DDIT3 gene expression suggests that Agomelatine has a neuroprotective mechanism that is independent of amyloid burden. Future studies will analyze neuronal loss via NeuN staining to determine if AGO prevents neuronal loss, thus supporting its ability to mitigate cognitive deficits in the TgF344‐AD rat model.

